# Progress in Surgery

**Published:** 1899-09-23

**Authors:** 


### Progress in Surgery.

### GENERAL SURGERY.

(*Continued from page* 425.)

**Fractures and Dislocations—Upper Extremity.** — An unusual case of simultaneous dislocation of both ends of the left clavicle in a lad, aged 17, is recorded by F. Brush.[6] While playing at football the boy was violently thrown, and fell upon a comrade. The posture was striking. With his right hand he held the left arm flexed, the hand supinated, and the elbow close to the side. His left shoulder was depressed and rotated forwards, while the head was turned to the injured side until the chin almost rested upon the shoulder. The outer end of the clavicle was displaced upwards and slightly overlapped the acromion process. The inner end was dislocated forwards towards the median line, resting on the anterior upper border of the sternum. A few sternal fibres of the sterno-mastoid which were not torn away were stretched tightly over the neck of the dislocated bone. By means of a large axillary pad, as a fulcrum, and pressure directly on the end of the bone, the outer dislocation was easily reduced and showed but slight tendency to recur. By prizing the shoulder outwards over a larger axillary fulcrum, combined with backward rotation of the shoulder and direct pressure on the dislocated bone, the sternal end was replaced. The bone was retained in good position by a combined Sayre and Velpeau dressing with a pad in axilla. No anæsthetic was used. The patient was discharged cured in the third week. Three cases of dislocation of the inner end and seven of the outer end of the clavicle are included in J. E. Platt's[7] collection of cases of injury of the upper extremity mentioned below, and are well worth perusal in this connection. One of the former occurred in a lad aged 18 years. Stanley Boyd[8] gives an interesting skiagram of a very oblique fracture of the upper fourth of the right humerus in a boy who had fallen off a scaffold ten months previously. After treatment in the country the upper end of the lower fragment projected beneath the skin over the deltoid, This unsatisfactory condition was probably due to the coexisting fracture of the outer part of the lower end of the humerus, which involved the elbow joint. There was an inch shortening, but the movements of the shoulder joint were free. The projecting part of bone was chiselled off through an incision along the anterior edge of deltoid, thus avoiding injury to the circumflex nerve. Two instances of ununited fracture of the humerus caused by the interposition of the musculo-spiral nerve between the fragments were recently brought before the Clinical Society by Mr. Clement Lucas.[9] The first case, a compound one, occurred in a man, aged 41, from the kick of a horse. He had been treated at a local infirmary by a rectangular splint. Some sloughing of the skin occurred, and three months later wire was applied, as union had not taken place. When seen three months afterwards there was a false joint at the junction of the upper two-thirds with the lower third of the humerus. The elbow was fixed in a semiflexed condition; the finger and wrist were also flexed and fixed. Loss of sensation existed over the distribution of the radial nerve. After treatment by massage, electricity, &c., an incision was made on the back of the arm and the musculo-spiral nerve traced up to where it was found engaged between the fragments. The wire encircling the fragments was found to include the fibrous extension of the nerve. The bone was again resected and wired; and the nerve, being cleared for about two inches above and below, was also resected, about an inch of the fibrous part being cut away and the ends united by means of sterilised silk. The bone subsequently firmly united, but the paralysis of the nerve remained. The second case was in a man aged 30, who came under Mr. Lucas' care for ununited fracture and musculo-spiral paralysis five months after the injury—a direct blow from the shaft of a cart. Severe pain down the back of the forearm and the outer side, extending to the thumb and forefinger, still existed, when Mr. Lucas, by an antero-external incision, traced up the musculo-spiral nerve to where it was engaged between the fragments. The nerve was detached, resected, and united by silk, and the fragments of bone were resected and united by a screw and an encircling wire, primary union following, and the patient left the hospital at the end of the third week. A month later good union occurred, and, though he had a useful limb, the power of extension at the wrist had not been recovered. In his address on surgery at the annual meeting of the British Medical Association, Mr. Edmund Owen[10] again drew attention to ununited fracture in children, and asks why it is of comparatively frequent occurrence in the tibia and fibula. He considers that want of rest, as in the adult, may account for a few cases, yet that in many others the neglect of proper treatment cannot explain the fact that operative treatment so often ends in amputation. He suggests that leading up to the fracture there is some subtle disturbance in the anterior cornu of the grey crescent of the cord, inhibiting the due nutrition of the bone, and rendering it weak and friable, subsequently interfering with repair, and eventually frustrating the best endeavours of the operating surgeon. He would thus regard the condition as one allied to the molecular disturbance of the crescent which entails infantile paralysis, but one expending its influence upon bone rather than muscle. J. E. Platt[11] continues his valuable analysis of 700 cases of fracture and dislocation of the upper extremity (see The Hospital, April 1st, 1899), and finds among them 60 cases of fracture of the lower end of the humerus, as follows: Separations of the lower epiphysis, 13; transverse fractures near the elbow joint, 7; T-shaped fractures, 3; oblique fractures of the external condyle into joint, 20; oblique fractures of the interior condyle into joint, 7; separations of the interior epicondyle, 9; fracture of the capitellum and trochlea, 1. The complications of separation of the lower epiphysis of the humerus he enumerates as (1) injury to the brachial artery; (2) extensive laceration of the ligaments of the elbow joint; (3) dislocation of the elbow; (4) laceration of the brachialis anticus; (5) dislocation of the biceps tendon; (6) extensive separation of the periosteum from the diaphysis; (7) pressure upon the nerve trunks; (8) gangrene; (9) compound separation of the epiphysis. Out of the 13 cases mentioned all, with two exceptions (both aged nine years), were below seven years of age. But it is evident that, from the anatomical condition of the lower end of the humerus at this early period, the second complication described, viz., "very extensive laceration of the lateral ligaments," can only occur under most exceptional circumstances, for the whole cartilaginous lower end of the humerus at this time, if separated by injury, still retains all its connections with the radius and ulna, the elbow joint being intact. Two skiagrams showing partial separation of the epiphysis with fracture of the diaphysis, taken two months after the injury, are of interest; while a third, of transverse fracture seven months after the accident, shows marked backward displacement of the lower fragment. Three other skiagrams display cubitus varus following separation of the lower epiphysis. Transverse fracture of the lower end of the humerus above the epicondyles with displacement forwards of the lower fragment is probably not so infrequent an injury in children as has hitherto been supposed, if we may judge from several excellent skiagrams of this lesion recently (August) published by Dr. Mackintosh,[12] of Glasgow, in his Skiagraphic Atlas of Fractures and Dislocations. As to the important subject of age, the nine cases of separation of the internal epicondyle of the humerus recorded by Platt[13] all were from 10 to 16 years, a fact which agrees very closely with the experience of other observers. Hutchinson says that in 33 cases of this injury which he collected the age ranged from eight to 18 years. Of 46 cases collected by Poland, in which the exact age of the patient was known, 34 were between 10 and 16 years old. True fracture of the epicondyle occasionally occurs in adults, although it is possible that in some of these cases the injury may be separation of an epiphysis, the union of which with the diaphysis has been delayed. Also among Platt's 700 cases of fracture and dislocation of the upper extremity 342 were of the radius and ulna; of these 16 were of the upper ends of these bones, viz., olecranon process, 12; coronoid process, 3; head of radius, 1. Out of the 12 fractures of the olecranon, it should be noted that 2 were said to have occurred in children, one aged five and the other aged seven. Two additional cases of compound fracture of the olecranon in young adults are quoted; in one primary excision of the joint was performed; in the other, the joint was carefully cleansed, and the edges of the wound, after paring, were brought together, there being little separation of the fragments. Both patients made a good recovery. Platt gives a skiagram of fracture of the head of the radius in a boy aged 17 years, complicated with fracture of the outer condyloid portion of the humerus and of the olecranon, in which good recovery ensued three years after the injury. Fractures of the head and neck of the radius are probably more frequent than is generally supposed. Platt then gives an analysis of 132 cases of fracture of the shafts of the radius and ulna in children and adults, many of which are full of interest. The same writer also records[15] 144 cases of fracture of the lower ends of the radius and ulna, including 36 epiphysial separations of the radius alone, six of epiphysis of radius and ulna, one of ulnar epiphysis alone (but in this instance the symptoms quoted are too meagre to afford an accurate diagnosis of so rare a lesion), 92 fractures of the radius alone, seven of radius and ulna, and two of ulna alone. Since the advent of the Röntgen process it has been conclusively proved that fracture of the ulnar styloid process is of very common occurrence in Colles's fracture. Several excellent skiagrams in the second edition of Walsh's[16] "Röntgen Rays in Medical Work," just (August) published, bear out the truth of this statement.

Dr. J. B. Roberts,[17] in a paper before the Philadelphia Academy of Surgery, emphasises the facts that fractures of the base of the radius must be reduced, if deformity, protracted convalescence, prolonged rigidity of joints, and pain are to be avoided, and that a compress, applied over the deformity due to impacted and unreduced fragments, is a futile substitute for the muscular force to be exerted on first seeing the injury. He attributes the neglect to reduce the fragments to the teaching of twenty years ago, when the pathology of the lesion was misunderstood. The importance of immediate and as perfect reduction as possible, he says, even if it is necessary to use an anæsthetic for its accomplishment, cannot be over-estimated; and it must be borne in mind that what appears to be slight malposition at first will steadily and surely pronounce itself as the swelling subsides, and, when it is too late to reduce it by either sudden or gradual means, an ugly, fork-shaped deformity will frequently present itself if complete reduction has not been performed at the beginning. The fragments can usually be brought into accurate apposition by direct and forcible pressure and counter-pressure with the thumbs and index fingers, and, once in place, very rarely show any tendency to become disarranged.

[6] The Post Graduate, N.Y., 1899, p. 31. [7] Med. Chron., Mar., 1899, p. 403. [8] Clin. Jour., Mar. 15, 1899, p. 342. [9] Lancet, April 22, 1899, p. 1094. [10] Brit. Med. Jour., Aug. 19, 1899, p. 449. [11] Med. Chron., Dec., 1898. [12] Skiagraphic Atlas of Fractures and Dislocations, 1899. [13] Med. Chron., Dec., 1898, p. 182. [14] Med. Chron., Jan., 1899, p. 257. [15] Ibid., Feb., 1899, p. 333. [16] Röntgen Rays in Medical Work, 1899. [17] Annals of Surgery, Feb., 1899, p. 272.

### OTOLOGY.

(*Continued from page 424.*)

**Sinus Thrombosis.**—Several cases of this complication of otitis have recently been recorded. Köller,[1] in relating a case, remarks upon the importance of operating early, and says that in cases in which the pyæmic condition has been allowed to last a week or longer, or in which pulmonic metastases are present, there is hardly a chance of recovery. Köller's case was that of a girl of 13, with history of double ear discharge for 10 years, dating from an attack of scarlet fever. She had had rigors daily for two weeks. Though there was pain and discharge from both ears the symptoms on the right side were the more marked, and there was a hard cord felt in the line of the jugular vein there. Pleuritic friction was present on the left side. The antrum was opened and found full of pus, then the sinus was exposed, and pus drawn out with a syringe. The jugular vein was next ligated, the upper part opened, and a septic thrombus removed. Then the sinus was scraped. She died the next day. In this case there was tenderness along the course of the jugulars on both sides, which, according to Macewen, is not infrequently the case, and is attributable to venous communications with the other side. The tenderness in the right occipital region led to the correct diagnosis. According to Koerner's statistics the results of these operations are better if the jugular vein is ligated before the sinus is opened and emptied. J. Dunn[2] records a case in which a patient, the subject of repeated attacks of otitis, had a pyæmic temperature, and extreme cellulitis of the scalp. The mastoid was found to be transformed into a cheesy mass, and to communicate with an extra-dural abscess, and with the interior of the lateral sinus. The latter was scraped, in the direction of the bulb, until a free flow of blood took place. The jugular vein was not ligatured. Recovery eventually followed. F. Whiting,[2] writing on this subject, describes three clinical stages. In the first there is a thrombus, parietal or complete, not having undergone disintegration, with moderate pyrexia, rigors being slight or absent. If recognised this should be operated upon, as all thrombi are probably infective. In the second stage the thrombus has undergone disintegration, and therefore there are pronounced fluctuations of temperature. In the third stage there are embolic metastases, terminating usually in septic pneumonia or enteritis. The lungs are attacked one and a half times oftener than the combined other structures of the body. The writer advocates complete removal of the thrombus in all positions. Air may be drawn into the jugular if there is respiratory pulsation below the clot, between it and the jugular bulb, and if the thrombus be removed too suddenly. The difficulty of diagnosing an obstruction low down in the bulb, it is pointed out, may be overcome by observing if the sinus above it fills again from below after emptying it towards the torcular by finger pressure, and emptying the jugular from above downwards similarly. In such a case the jugular may be ligated low down, and again near the base of the skull, and the portion between the ligatures removed, and the bulb washed out from above. Dr. J. E. Sheppard[3] records another case of thrombosis. It was successfully treated. The patient was a lady of 20, who for ten days had left earache, otorrhœa, and rigors. The drum had perforated. There was no external evidence of mastoid disease except tenderness over the region of the emissary vein. The mastoid cells contained pus. The sinus was exposed, and had a layer of pus over it. Four days later, the temperature being 105 deg., the sinus was opened and thrombotic material and pus cleared out. Seventeen days later the left jugular was tied, and two inches and a half removed, free from clot. The sinus was explored downwards towards the jugular bulb and pus evacuated, and backwards, with Rongeur forceps, to within an inch of the torcular. It was slit up nearly to this point, and thrombotic material removed till blood flowed. The patient made a rapid recovery. That the first rigor should have been so soon as six days after the onset of earache is remarkable. There was an extreme degree of leucocytosis before the last operation.

[1] Med. Rec., Feb., 1899. [2] Jour. of Laryngol., Rhin., and Otology, Mar., 1899. [3] Ditto, June, 1899.

